# Supplementary material for: Subjective Deterioration of Physical and Psychological Health during the COVID-19 Pandemic in Taiwan: Their Association with the Adoption of Protective Behaviors and Mental Health Problems
Source: Int J Environ Res Public Health. 2020 Sep 18;17(18):6827. doi: 10.3390/ijerph17186827 (PMC7559552; doi:10.3390/ijerph17186827)
Supplement: Supplementary file 1 [file ijerph-17-06827-s001.pdf]

Supplementary Table S1

Measures Used in This Study

Measure	Item	Response scale	Dichotomous scale
Self-reported physical health status	How is the state of your physical health compared with other people before the COVID-19 pandemic (in the recent week)?	1 = much worse, 2 = mildly worse, 3 = the same, 4 = mildly better, 5 = much better	
Self-reported psychological health status	How is the state of your psychological health compared with other people before the COVID-19 pandemic (in the recent week)?	1 = much worse, 2 = mildly worse, 3 = the same, 4 = mildly better, 5 = much better	
Cognitive construct of health beliefs	Item 1: What do you think are your chances of contracting COVID-19 over the next month compared with those outside your family?	1 = no chance of contracting COVID-19, 2 = very unlikely, 3 = unlikely, 4 = evens, 5 = likely, 6 = very likely, 7 = guaranteed to contract COVID-19	0 = low perceived susceptibility (score < 5)<br>1 = high perceived susceptibility (score ≥ 5)
	Item 2: How serious is COVID-19 relative to SARS?	1 = much less serious, 2 = less serious, 3 = similar, 4 = more serious, 5 = much more serious	0 = perceived harm of COVID-19 less than or similar to SARS (score < 4)<br>1 = perceived harm of COVID-19 more than SARS (score ≥ 4)
	Item 3: Do you think you have sufficient knowledge and information on COVID-19?	0 = Insufficient, 1 = sufficient	

	Item 4: How confident are you that you can cope with COVID-19?	1 = not confident at all, 2 = mildly unconfident, 3 = fair, 4 = confident, 5 = very confident	0 = low confidence in coping with COVID-19 (score < 3)<br>1 = high confidence in coping with COVID-19 (score ≥ 3)
Affective construct of health beliefs	Please rate how worried you are about COVID-19:	Scores ranged from 1 to 10 (1 = very mild, 10 = very severe)	0 = low worry about COVID-19 (score < 6)<br>1 = high worry about COVID-19 (score ≥ 6)
Perceived social support<br>(total score of items 1 to 3)	In the past 7 days, were you satisfied with the support from your 1) family, 2) friends, and 3) colleagues or classmates?	0 = not satisfied at all, 1 = mildly unsatisfied, 2 = fair, 3 = satisfied, 4 = very satisfied	
Adoption of protective behaviors	In the past week, did you 1) avoid going to crowded places, 2) wash your hands more often, and 3) wear a mask more often?	0 = no, 1 = yes, but not due to COVID-19, 2 = yes, due to COVID-19	0 = no (score ≤ 1)<br>1 = yes (score = 2)
General anxiety	Agreement on 10 statements: I feel rested /content/comfortable/relaxed/pleasant/anxious/nervous/jittery/"high strung"/over-excited and "rattled"	1 = not at all, 2 = sometimes, 3 = moderately so, 4 = very	0 = low general anxiety (total score ≤ median score [23])<br>1 = high general anxiety (total score > median score [23])
Sleep problems	In the past week, did you have sleep problems?	0 = never, 1 = mild, 2 = moderate, 3 = severe, 4 = extremely severe	0 = no sleep problem (score = 0)<br>1 = sleep problems (score > 0)
Suicidal ideation	In the past week, did you ever have suicidal thoughts?	0 = never, 1 = mild, 2 = moderate, 3 = severe, 4 = extremely severe	0 = no suicidal ideation (score = 0)<br>1 = suicidal ideation (score > 0)
